# Cross‐linking disulfide bonds govern solution structures of diabodies

**DOI:** 10.1002/prot.26509

**Published:** 2023-06-28

**Authors:** Barbara A. Math, Franz Waibl, Leonida M. Lamp, Monica L. Fernández‐Quintero, Klaus R. Liedl

**Affiliations:** ^1^ Institute of General, Inorganic and Theoretical Chemistry, and Center for Molecular Biosciences Innsbruck (CMBI) University of Innsbruck Innsbruck Austria

**Keywords:** diabodies, enhanced sampling, interdomain orientation dynamics, molecular dynamics simulations, novel antibody formats

## Abstract

In the last years, antibodies have emerged as a promising new class of therapeutics, due to their combination of high specificity with long serum half‐life and low risk of side‐effects. Diabodies are a popular novel antibody format, consisting of two *F*_v_ domains connected with short linkers. Like IgG antibodies, they simultaneously bind two target proteins. However, they offer altered properties, given their smaller size and higher rigidity. In this study, we conducted the—to our knowledge—first molecular dynamics (MD) simulations of diabodies and find a surprisingly high conformational flexibility in the relative orientation of the two *F*_v_ domains. We observe rigidifying effects through the introduction of disulfide bonds in the *F*_v_–*F*_v_ interface and characterize the effect of different disulfide bond locations on the conformation. Additionally, we compare *V*_H_–*V*_L_ orientations and paratope dynamics between diabodies and an antigen binding fragment (Fab) of the same sequence. We find mostly consistent structures and dynamics, indicating similar antigen binding properties. The most significant differences can be found within the CDR‐H2 loop dynamics. Of all CDR loops, the CDR‐H2 is located closest to the artificial *F*_v_–*F*_v_ interface. All examined diabodies show similar *V*_H_–*V*_L_ orientations, *F*_v_–*F*_v_ packing and CDR loop conformations. However, the variant with a P14C‐K64C disulfide bond differs most from the Fab in our measures, including the CDR‐H3 loop conformational ensemble. This suggests altered antigen binding properties and underlines the need for careful validation of the disulfide bond locations in diabodies.

## 1 INTRODUCTION

Antibodies play a central role in the adaptive immune system, as they provide a highly specific response to foreign molecules.^1^ The interaction with the antigen occurs at one of the two N‐terminal variable domains (*F*_v_) of the antigen binding fragments (Fabs). Each *F*_v_ is formed by a heavy (*V*_H_) and a light chain (*V*_L_),^2^ the relative packing between *V*_H_ and *V*_L_ is expected to have an impact on the antigen binding properties.^3^, ^4^, ^5^, ^6^, ^7^

The high affinity and specificity of antibodies are achieved by three hypervariable loops on each *V*_H_ and *V*_L_, referred to as Complementarity Determining Regions (CDRs).^8^ Among the six CDR loops, the CDR‐H3 loop has the largest variety in sequence and structure.^9^, ^10^ However, all CDR loops show transitions between different CDR loop states in the micro‐ to millisecond timescale.^11^ Different conformations result in altered binding properties, thus making sequence‐based predictions more challenging.^8^, ^11^, ^12^, ^13^, ^14^, ^15^

Antibody‐based drugs are the fastest growing drug class,^16^ given their ability to bind specific molecules with high specificity and affinity, as well as their low risk of side effects.^17^ They can further be used as targeted delivery system in antibody‐drug conjugates.^18^, ^19^ In 2023, over 120 antibody‐based drugs were approved by the FDA.^20^ However, the use of conventional monoclonal antibodies (mAbs) is limited due to the high molecular weight and subsequently low diffusion rate,^21^ as well as their limited ability to cross cell membranes and the blood–brain barrier.^22^, ^23^ To overcome this limitation, numerous smaller variants have been proposed.^24^ Diabodies combine a smaller size compared to conventional IgGs with the ability to create bispecific binders and offer different mechanisms of action by binding to a target and a T‐cell at the same time.^24^, ^25^, ^26^, ^27^, ^28^, ^29^ The smaller size comes at the cost of an Fc region, which can be important for interactions with the immune system, but also cause immunogenic reactions.^19^

## 2 DIABODY STRUCTURE

**FIGURE 1:**
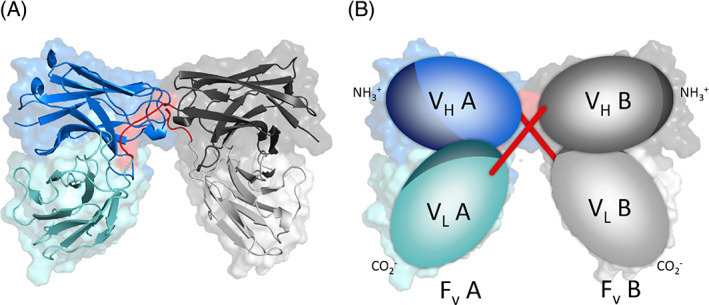
(A) Cartoon representation of the PDB structure 5GRW. (B) Schematic representation of the diabody structure. In both subplots, the *F*_v_ of side A is colored blue, while the *F*_v_ of side B is gray. For both *F*_v_s the *V*_H_ is shown in a darker color compared to the *V*_L_. The CDR loops are represented with a darker blue or gray. The interdomain linker is represented in red.

Diabodies were first designed by Hollinger et al. in 1993.^30^ They are constructed by connecting *V*_H_ and *V*_L_ fragments with a linker consisting of three to twelve residues, resulting in the formation of two interchain *F*_v_ domains.^31^ Diabodies can be arranged in *V*_H_–*V*_L_ and *V*_L_–*V*_H_ orientation,^32^ a schematic representation of a *V*_H_–*V*_L_ structure is given in Figure 1.

The most commonly used linker consists of a five amino acid (Gly_4_Ser) fragment,^31^ which is flexible, protease‐resistant and does not interfere with domain folding.^33^ Alternatively, the linker sequence can be optimized by phage display.^34^, ^35^

Diabodies offer multiple applications not only in biological drug discovery, but also in structural biology and protein nanotechnology.^36^ They show a higher binding affinity and structural stability compared to single chain *F*_v_ (scFvs).^37^ Another strength of diabodies is their bivalency, which can be used to induce dimerization of target proteins^38^, ^39^ and allows for the development of bispecific diabodies^40^, ^41^, ^42^, ^43^, ^44^, ^45^, ^46^: Several formats have been developed, including Tandem Diabodies,^47^ BiTE,^48^ and DARTs.^49^, ^50^ Among these, the DART Blinatumomab by Amgen was approved in 2014 by the FDA.^51^, ^52^, ^53^, ^54^

## 3 DIABODY STRUCTURAL DIVERSITY

The relevance of relative diabody *F*_v_–*F*_v_ orientations is highlighted in a study by Moraga et al.^55^: several monospecific diabodies, directed against the erythropoietin receptor, were generated. Different levels of pathway activation were detected, depending on the conformation of the diabodies and subsequent differences in the relative proximity and orientations of the two extracellular receptor molecules.^55^

The structural diversity of diabodies was previously investigated with different methods. An early experimental approach describing diabody conformations was developed by Lawrence et al. in 1998^56^: Both epitopes of the diabody were experimentally complexed with an anti‐idiotype Fab each, thus extending the “arms” of the diabodies. This allowed the determination of a diabody angle in electron micrographs.^56^ A more recent study by Kwon et al.^36^ compared the crystal structure conformations of 17 diabody structures by describing the *F*_v_ interface. The geometries of the diabodies were described with relative conformations of four conserved tryptophan residues (W35 on each of the *V*_H_ fragments and W35 on *V*_L_).^36^

In these studies, a large variability in the relative *F*_v_–*F*_v_ orientations was observed,^36^, ^56^ in line with the highly flexible elbow angle^57^ in antibodies.^58^, ^59^, ^60^ The high structural variability was attributed to the relatively small interaction interface between the two *V*_H_ domains, forming no hydrogen bond network or hydrophobic patch.^36^ Moreover, arginines in the artificial *V*_H_–*V*_H_ interface (common at position 83 in Kabat numbering scheme^61^) are hypothesized to additionally increase structural diversity by charge repulsion.^38^ On the other hand, the introduction of disulfide bonds is expected to introduce rigidity in the binding site orientations, to control the relative geometry and distance of the bound proteins, and prevent chain shuffling events.^38^

In this study, we characterize the relative *F*_v_–*F*_v_ orientations of a set of four diabodies with nearly identical sequences, differing solely in the location of stabilizing disulfide bonds and the residue at position H83. We find a significant reduction of the conformational flexibility and a shift in the preferred relative orientations, in dependence of the disulfide bridges. Extensive sampling in combination with the bias‐exchange enhanced sampling technique allows us to characterize loop conformations in solution. We compare the loop conformations of the different diabody variants to a Fab fragment with identical CDR and *Fv* sequences and find a high overlap. The highest differences are observed in the CDR‐H2 and CDR‐H3 loops. The variant with a P14C‐K64C disulfide bond shows a conformational shift in the loop conformations, as well as in the relative *V*_H_–*V*_L_ orientations, suggesting altered antigen binding properties.

## 4 METHODS

### 4.1 Dataset

In this study, a dataset consisting of four diabodies (DAb) and one Fab fragment was used. The sequences of the *F*_v_s were identical in all systems, besides the amino acid at position H83 (Kabat numbering) and stabilizing disulfide bonds (details in Table 1 and structural representations in Figure S1). H83 lies directly in the artificial *F*_v_–*F*_v_ interface. Kim et al. hypothesize that the amino acid at this position has a major impact on the diabody flexibility.^38^

**TABLE 1:** Overview on the dataset used.

	PDB code	Antibody format	Amino acid at position H83	Location of SS‐bridges	
Fab	2V7N	Fab	Arg		62
DAb_R83F	5GRW	Diabody	Phe		38
DAb_E85C	5GRX	Diabody	Thr	E85C	38
DAb_D61C‐S113C	5GRY	Diabody	Thr	D61C, S113C	38
DAb_P14C‐K64C	5GRZ	Diabody	Thr	P14C, K64C	38

*Note*: Amino acid positions are numbered in Kabat scheme.

The original Fab binds to the sodium citrate symporter CitS of *Klebsiella pneumoniae*, and was generated by phage display.^62^ CitS is a membrane protein that facilitates the symport of citrate and sodium ions, allowing the bacteria to use citrate as sole source of carbon and energy.^63^

### 4.2 Molecular dynamics simulations

#### 4.2.1 Structure preparation and equilibration

The starting structures for the simulations, as listed in Table 1, were downloaded from the Protein Data Bank (PDB)^64^ and automatically protonated to a physiological pH of 7.4 with the “Protonate 3D” tool^65^ in MOE, version 2018.01.^66^ After visual inspection, the resulting protein structures were solvated in a cubic box of TIP3P water^67^ using the tleap program from AmberTools 19.^68^ The minimal wall distance was set to 10 Å. The solvated proteins were minimized and equilibrated in the NpT ensemble according to a procedure previously developed in our group,^69^ using the GPU implementation^70^ of PMEMD in Amber18^68^ with the ff14SB force field.^71^

#### 4.2.2 Molecular dynamics simulations

Molecular dynamics (MD) simulations of the equilibrated structures were performed in an isothermal‐isobaric (NpT) ensemble under periodic boundary conditions.^72^ The temperature was set to 300 K using the Langevin thermostat.^73^ For pressure adjustment at 1 bar, the Berendsen barostat^74^ was used. The simulation time step was set at 2 fs in all simulations. Bonds involving hydrogen atoms were constrained using the SHAKE^75^ algorithm. For the treatment of long range coulombic interactions, the Particle Mesh Ewald (PME)^76^ method was used with a cutoff radius of 8 Å.

#### 4.2.3 Bias‐exchange simulations

To enhance the sampling of the conformational space for the CDR loops, well‐tempered bias‐exchange metadynamics^77^, ^78^, ^79^, ^80^ simulations were performed. In this enhanced sampling technique, several metadynamics^77^, ^78^ simulations are run in parallel, each one sampling another predefined collective variable (CV). We chose a linear combination of the ψ backbone angles' sines and cosines of each individual CDR loop as CVs, resulting in 12 replicas in case of the diabodies and 6 replicas in the case of the Fab. This choice of collective variable has previously been reported to capture conformational transitions between antibody CDR loop conformations comprehensively.^10^, ^81^ Exchanges between the biases of the different replica were attempted every 2000 simulation step, according to a Metropolis scheme.

The simulations were performed using GROMACS 2020^82^, ^83^ with the PLUMED 2^84^, ^85^, ^86^ implementation. We simulated each replica for 500 ns, resulting in 6 μs biased simulations per diabody and 3 μs for the Fab fragment.

#### 4.2.4 Dihedral clustering and seed trajectories

To obtain unbiased ensembles from the bias‐exchange simulations, we clustered them and started short unbiased (seed) simulations from the cluster centers, as previously applied on antibody Fab fragments.^11^, ^13^ The simulations were clustered with a distance cutoff of 5.2 in case of the diabodies and a cutoff of 3.2 for the Fab, resulting in 77 (DAb_E85C) to 255 (DAb_R83F) clusters for the diabodies and in 288 clusters for the Fab fragment. Each of the cluster centers was used as starting point (seed) for unbiased simulations. The seed simulations were run for 100 ns using the GPU implementation^70^ of PMEMD in AMBER 20.

The clustering was performed with the average linkage hierarchical clustering algorithm in scipy.^87^ We performed a clustering of the CDR loop conformations in dihedral space since the clustering of all 12 loops in Cartesian space is highly influenced by the relative *F*_v_–*F*_v_ orientations. We chose a dihedral clustering similar to North et al.^15^ by adding up the cosines of the distances of all ψ and φ CDR backbone angles (see below equations).

$$d_{\theta_{i}\theta_{j}}=2\left(1-\cos\left(\theta_{i}-\theta_{j}\right)\right)$$

$$D^{A,B}=\sum_{r=0}^{N}\left(d_{\phi_{r}^{A}\phi_{r}^{B}}+d_{\psi_{r}^{A}\psi_{r}^{B}}\right)$$

### 4.3 Analysis

#### 4.3.1 Diabody dihedral

The diabody dihedrals were calculated, as described by Kwon et al.,^36^ by determining the dihedrals between the Cα atoms of the conserved W36 (*V*_H_)/W35 (*V*_L_) residues. The analysis was carried out with an in‐house python script using mdtraj.^88^

#### 4.3.2 Diabody angle

For the calculation of the diabody angles, the angle between the *F*_v_ pseudo dyad axes for the two domains was determined with a python^89^ script.

The pseudo dyad axis was determined by first calculating the rotational axis between the *V*_H_ and *V*_L_ domains (with TM‐align^90^) and taking the eigenvector associated to the first eigenvalue. Subsequently, the angle between the two pseudo dyad axes was determined. To avoid ambiguous angles, all pseudo dyad vectors were directed from the C‐ to the N‐ terminus of the *F*_v_ domains, before calculating the angles.

#### 4.3.3 Sphere projections

For the calculation of the sphere projections, one *F*_v_ domain of each simulation frame was aligned to a reference *F*_v_ structure (PDB file provided in the Supporting Information). The reference structure consists of the β‐sheets of an *F*_v_, centered on the hinge residues. Additionally, the rotational axis between the *V*_H_ and *V*_L_ domains is oriented in z‐direction.

**FIGURE 2:**
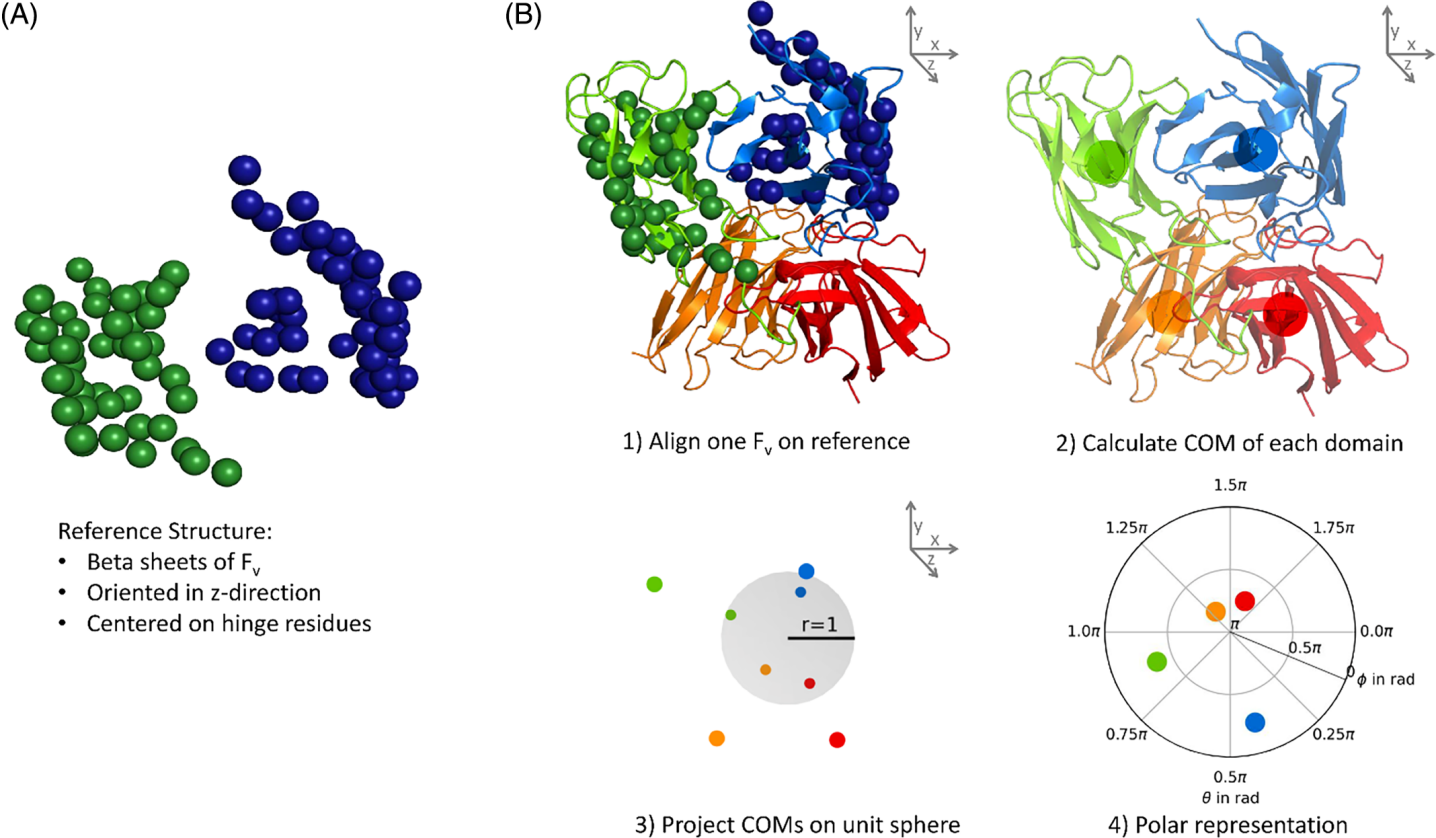
Graphical representation of the diabody projection on spherical coordinates. (A) Representation of the reference structure, consisting of the Cα atoms of the β–sheets. Atoms of the *V*_H_ and *V*_L_ domains are colored in blue and green respectively. (B) Calculation of the spherical representation. (1) One of the two *F*_v_s is aligned on the reference structure. (2) The center of mass (COM) for each domain is calculated. (3) The COMs are projected on a unit sphere in Cartesian coordinates. (4) The projected COMs for both *F*_v_s are represented in polar coordinates.

The centers of mass (COM) of each *V*_H_ and *V*_L_ domain are then projected on a unit sphere and represented in 2D as polar plot with *φ* as radius and *θ* as circumference. The statistics are enhanced by calculating two sphere projections from each conformation: both *F*_v_s are aligned on the reference sequentially. A graphical overview on the process is given in Figure 2.

#### 4.3.4 *V*_H_–*V*_L_ orientations

The *V*_H_–*V*_L_ orientations of the diabodies and Fab fragment with nearly identical sequence were analyzed with the orientation of cylindrical domains (OCD) tool.^91^ This tool describes the orientation of two cylindrical domains with six measures, four angles, a distance, and a torsion. We chose the β‐sheet Cα atoms as mask for the calculation and used the same reference structure as for the sphere projection (Section 4.3.3 ).

#### 4.3.5 Principal component analysis

We conducted a principal component analysis (PCA) on each of the CDR loops (six loops per *F*_v_) individually with the pyEMMA2 python package^92^ using the backbone torsions as features.

#### 4.3.6 Histograms

For diabody dihedral, diabody angle and *V*_H_–*V*_L_ orientations histograms were calculated with the numpy python package^93^ and smoothened with a Gaussian kernel using the curvefit function in scipy.^87^ The resulting curves were plotted with the matplotlib python package.^94^

## 5 RESULTS

### 5.1 Description of *F*_v_–*F*_v_ orientations in literature

The relative orientation of the two *F*_v_s in a diabody can be described as a dihedral between conserved residues in the *V*_H_ and *V*_L_ regions, as suggested by Kwon et al. in 2019.^36^ We applied this metric on extensive simulations of four diabody variants, differing in the location of stabilizing intramolecular disulfide bonds and the residue at H83. As described in the methods section, we performed well‐tempered bias‐exchange metadynamics simulations, clustered the simulations and ran unbiased molecular dynamics simulations on each cluster representative (seed simulations).

**FIGURE 3:**
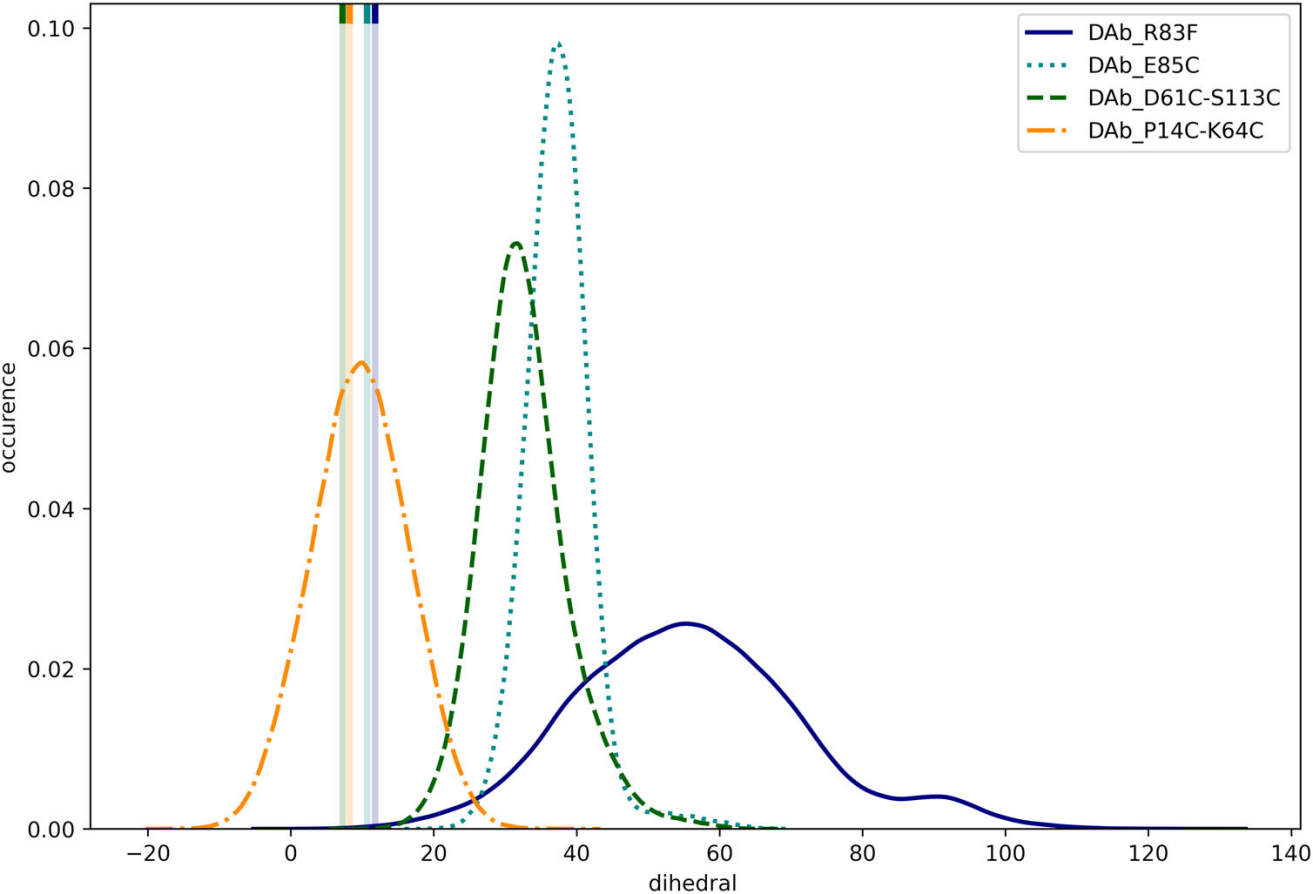
Relative occurrences of dihedrals in the seed simulations of the four diabodies. The dihedral of the corresponding crystal structure is shown as a vertical line.

We calculated the dihedral angle for each simulation frame of the seed simulations. As shown in Figure 3, most of the diabodies show a pronounced shift in the dihedral angle from the crystal structure to the preferred conformation in solution. The only exception is the DAb_P14C‐K64C system, which contains two additional disulfide bonds and stays very close to the dihedral angle of the crystal structure.

The broadest distribution is observed in the simulation of DAb_R83F, the only diabody without stabilizing disulfide bonds in this dataset. This simulation further shows a bimodal distribution, 4‐fold trajectory splitting confirmed this was not due to an equilibration effect. The narrowest distribution is observed in the DAb_E85C simulation, a diabody variant with one stabilizing disulfide bond.

To understand the structural implication of the dihedral, we show representative structures for DAb_R83F in Figure S2.

Another, earlier approach in the description of diabody conformation was developed by Lawrence et al. in 1998^56^: The angle between the diabody *F*_v_s was experimentally determined by electron micrographs.

**FIGURE 4:**
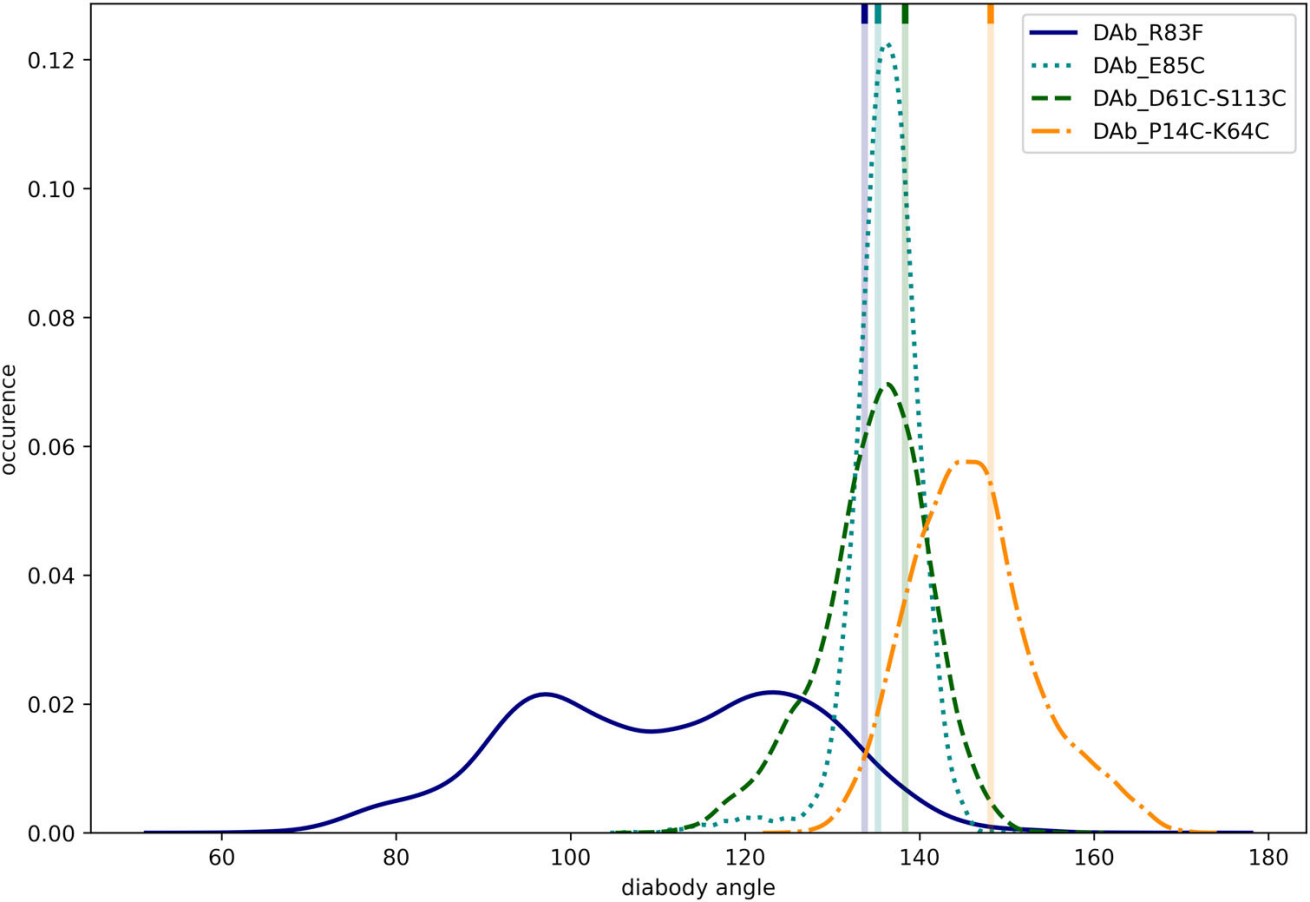
Relative occurrences of diabody angles in the seed simulations of the four diabodies. The angle of the corresponding crystal structure is shown as a vertical line.

We mimicked this method in silico by calculating the angle between the pseudo dyad vectors of both *F*_v_s, as described in the methods section. The probability distributions of these diabody angles in Figure 4 show similar trends as observed for the dihedrals (Figure 3). Again, the DAb_R83F simulation is bimodally distributed and shows the broadest spread. The narrowest distribution is seen for the DAb_E85C simulation, while the DAb_P14C‐K64C simulation shows a pronounced shift from the other diabodies in this metric. Combined with the dihedral angle, there are strong conformational changes in all investigated systems, as shown with representative structures of DAb_R83F in Figure S3.

### 5.2 Sphere projection

**FIGURE 5:**
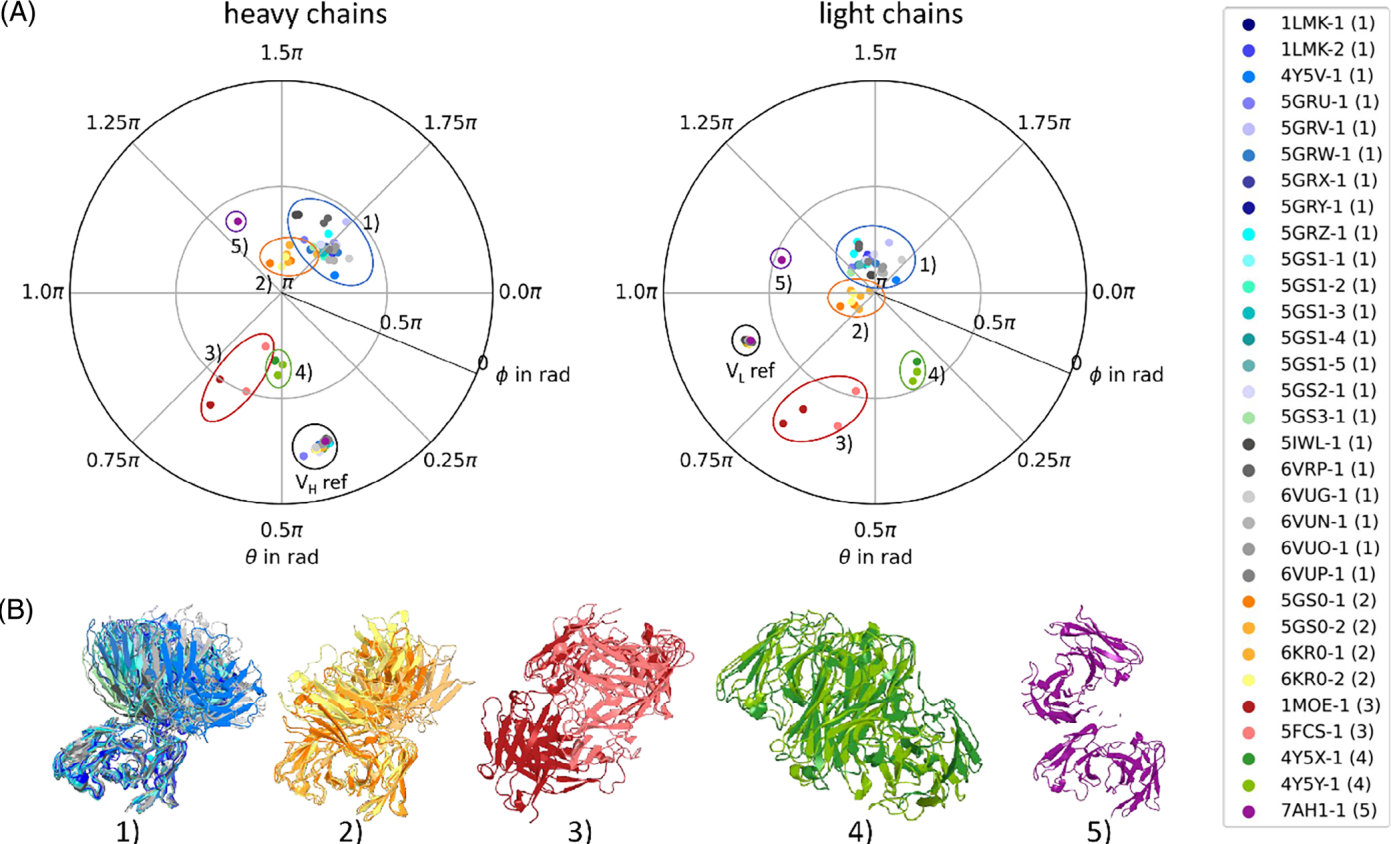
(A) Sphere representation for 31 crystal structures. In this representation, the centers of mass of *V*_H_ and *V*_L_ domains are projected on a sphere and represented as polar plot. Left panel: *V*_H_ domains. Right: *V*_L_ domains. In both subplots, the spherical coordinates of the reference structures are marked with a text label. Additionally, the crystal structures are grouped into five clusters, based on their locations in the sphere representations. (B) Cartoon representations of the crystal structures, grouped by the observed clustering in the sphere representation (A). All crystal structures are aligned on the same reference *F*_v_ domain and shown in the same orientation. Next to the plot, a legend with the color coding of the PDB codes is given, as well as their cluster in parenthesis.

We introduce a new description of diabody conformations by calculating the centers of mass (COM) for each *V*_H_ and *V*_L_ domain and projecting them on a unit sphere. When applying this metric on 31 crystal structures of the PDB,^38^, ^55^, ^64^, ^95^, ^96^, ^97^, ^98^, ^99^, ^100^, ^101^ we observe a clustering of the structures (Figure 5). We visually assigned the structures to five clusters, based on their *V*_H_ and *V*_L_ domains in our representation and find two major groups, cluster 1 with 22 structures and cluster 2 with 4 structures, respectively. Our representation suggests these clusters to be very similar: *V*_H_ and *V*_L_ domains show nearly identical *φ* values, but slightly differ in *θ*.

The two crystal structures 1MOE^96^ and 5FCS^97^ are the only structures of the dataset designed in a *V*_L_ to *V*_H_ linkage and form an own cluster in our representation (cluster 3).

Both crystal structures of cluster 4, 4Y5X and 4Y5Y,^55^ derive from the same publication and were shown to antagonize EPO pathway activation. Kim et al. hypothesized the remarkable difference to other known diabody structures to be caused by an arginine at position H83.^38^

In our representation, the structure with the PDB code 7AH1^101^ is very different to the others and can be classified as sole member of cluster 5. This structure was obtained by crystallizing a L19 diabody fragment, covalently bound to IL12. However, only the L19 diabody could be resolved.

**FIGURE 6:**
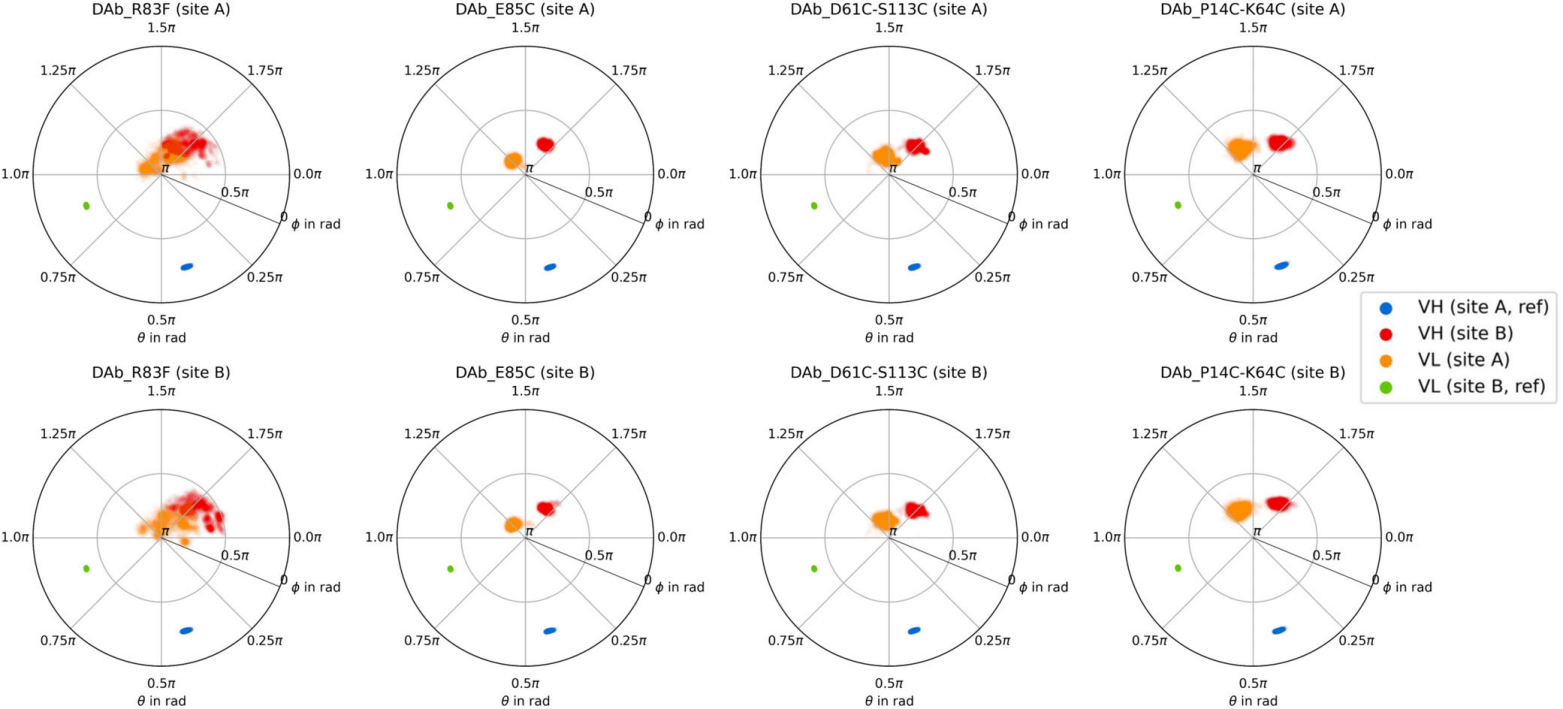
Sphere representation of the seed simulations. On each column, a different diabody is shown, for each row, one of the two identical *F*_v_s of the diabody was aligned to the reference *F*_v_. In each subplot, the *V*_H_ and *V*_L_ aligned to the reference structures (Figures 2 and 5) are represented in blue and green, the not aligned *V*_H_ and *V*_L_ domains in red and orange, respectively.

In Figure 6, the spherical projections for the seeded metadynamics simulations are shown. The conformational spaces for all four diabodies are restricted to a small area and lie within the region defined as cluster 1 of the crystal structures (Figure 5). No transitions to other clusters are observed.

Of the investigated simulations, the one started from the crystal structure without disulfide bonds (DAb_R83F) shows the highest flexibility. Additionally, it is associated with a bimodal distribution. This observation is in line with the distributions of dihedrals (Figure 3) and diabody angles (Figure 4). As in the other measures, the DAb_E85C simulation shows the narrowest distribution, while the conformational space for the DAb_P14C‐K64C is slightly shifted. For all diabodies, a symmetric behavior is observed: Independently of the site being aligned to the reference, the distribution is similar.

### 5.3 Influence on *V*_H_–*V*_L_ conformations

Besides the description of inter‐*F*_v_ orientations, we also investigated the influence of the interdomain disulfide bridges on *V*_H_–*V*_L_ conformations in diabodies. We used the Orientation of Cylindrical Domains (OCD) tool.^91^ This tool describes the orientation in immunoglobulin domains, as found in antibodies, with six measures. We applied the method on each simulation frame of the seeded metadynamics simulations and compared the distributions to the simulation of a Fab fragment with an identical sequence.

**FIGURE 7:**
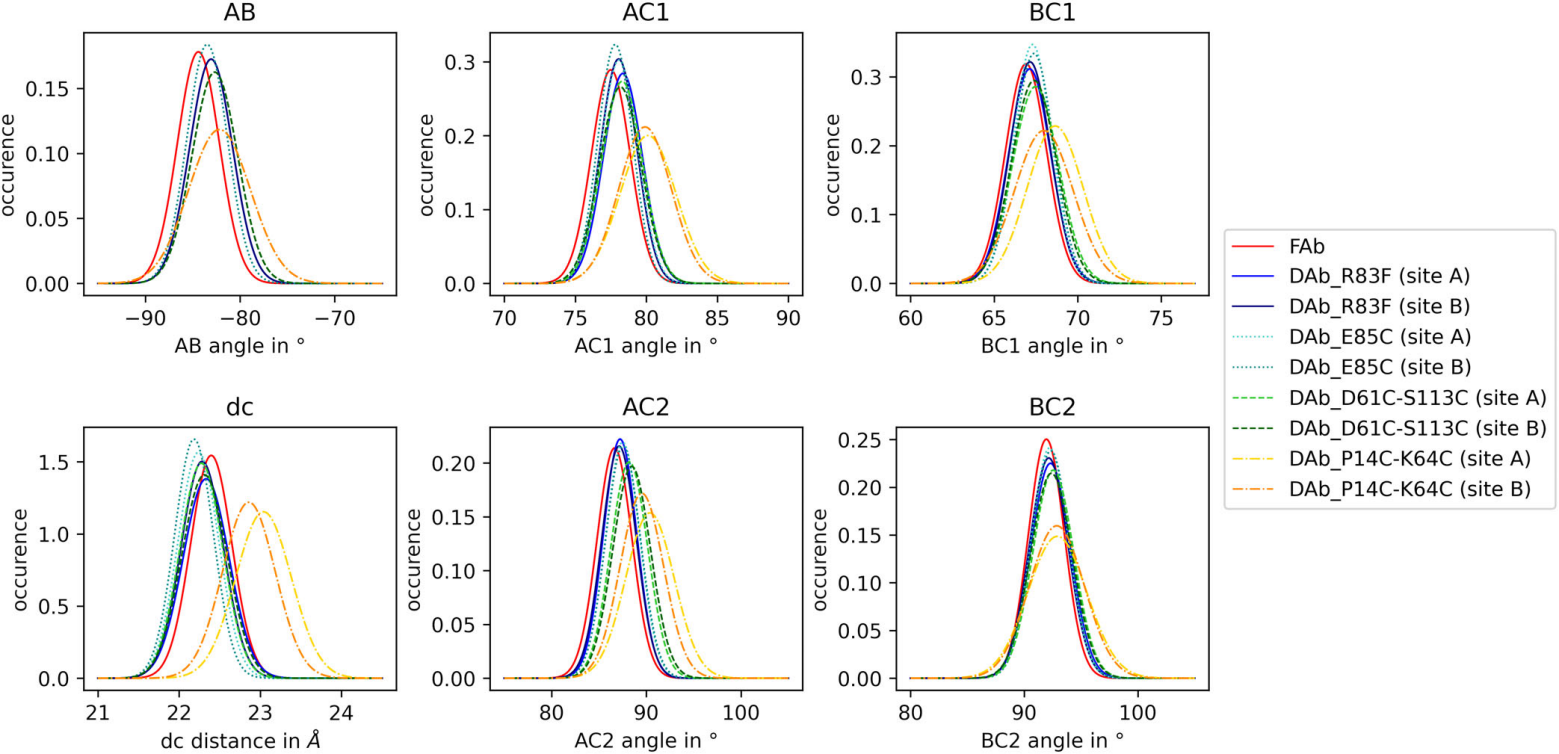
Distribution of the OCD measures for the seed simulations of the four diabodies and the antibody Fab. The histograms for the diabodies are colored in blue (DAb_R83F), teal (DAb_E85C), green (DAb_D61C‐S113C) and yellow (DAb_P14C‐K64C), the histogram for the antibody in red (Fab).

For all six measures, we find very similar distributions in the intra‐*F*_v_ orientations when comparing the diabody *F*_v_ simulations with the *F*_v_ from the Fab fragment. The highest differences to the Fab simulations, all well as to the other diabody simulations, are observed in the simulation of DAb_P14C‐K64C. The most significant shift is observed in the dc measure, indicating a less tightly packed *F*_v_ domain. However, also in the other measures, a shift is visible (Figure 7).

### 5.4 Influence on the CDR loops

The Complementarity Determining Region (CDR) loops are responsible for antigen recognition in both antibodies^8^ and diabodies.^30^ The interaction with the antigen is directly influenced by the CDR loop conformations. The geometric minimum in solution has been found to be close to the binding competent state for antibodies.^11^ Thus, we compared the conformational space for each of the six CDR loops in an unbiased ensemble between the four diabodies and the Fab fragment. We demonstrate the statistical significance of our analysis by analyzing both *F*_v_ domains in the system separately, expecting that they should give identical results in the case of perfect sampling due to the symmetry of the system.

We analyzed the backbone dihedrals for each individual CDR loop with a PCA. Overall, all diabody CDR loops show a very similar conformational space, compared to the Fab fragment. The L1, L2, L3 and H1 loop conformations are mainly restricted to a single minimum. However, the H2 and H3 loop show a more differentiated conformational landscape.

**FIGURE 8:**
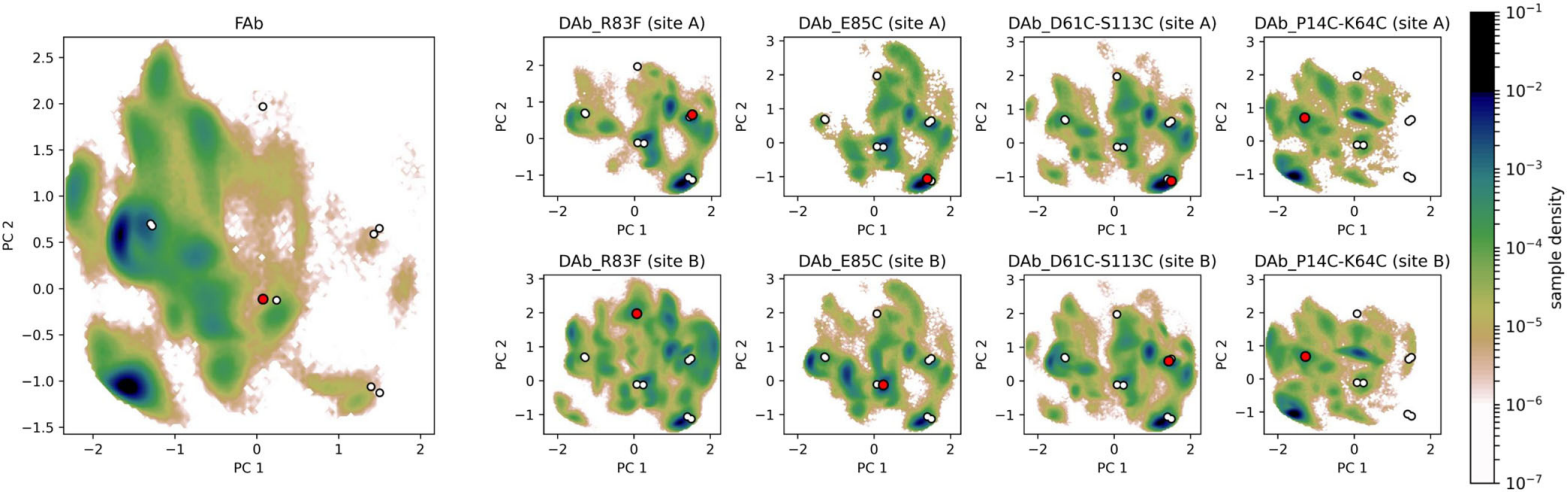
PCA plot on the CDR‐H2 dihedrals: The simulation of the Fab fragment on the left (Fab) is compared to the diabodies. For each diabody, the conformational space is shown for both *F*_v_ fragments (one site per row). In each subplot the red point shows the crystal structure the corresponding simulations was started from, while the white ones represent the other crystal structures.

As shown in Figure 8, the conformational space for the CDR‐H2 loop exhibits various energetical minima. The PCA plots suggest similar conformations between the DAb_P14C‐K64C simulation and the Fab fragment simulation (Fab) for the H2 loop: these two simulations cover a similar conformational space and show an energetic minimum in solution at the same geometries. The other diabody simulations (DAb_R83F, DAb_E85C, DAb_D61C‐S113C) share a very similar conformational landscape between each other but are different to the simulation of the Fab fragment: they poorly cover the conformational minimum of the Fab simulation. In most diabodies, the sites A and B are very similar, suggesting convergence of the simulations. The biggest differences are observed in DAb_R83F, the diabody without stabilizing disulfide bonds, where site B shows a more shallow landscape than site A.

**FIGURE 9:**
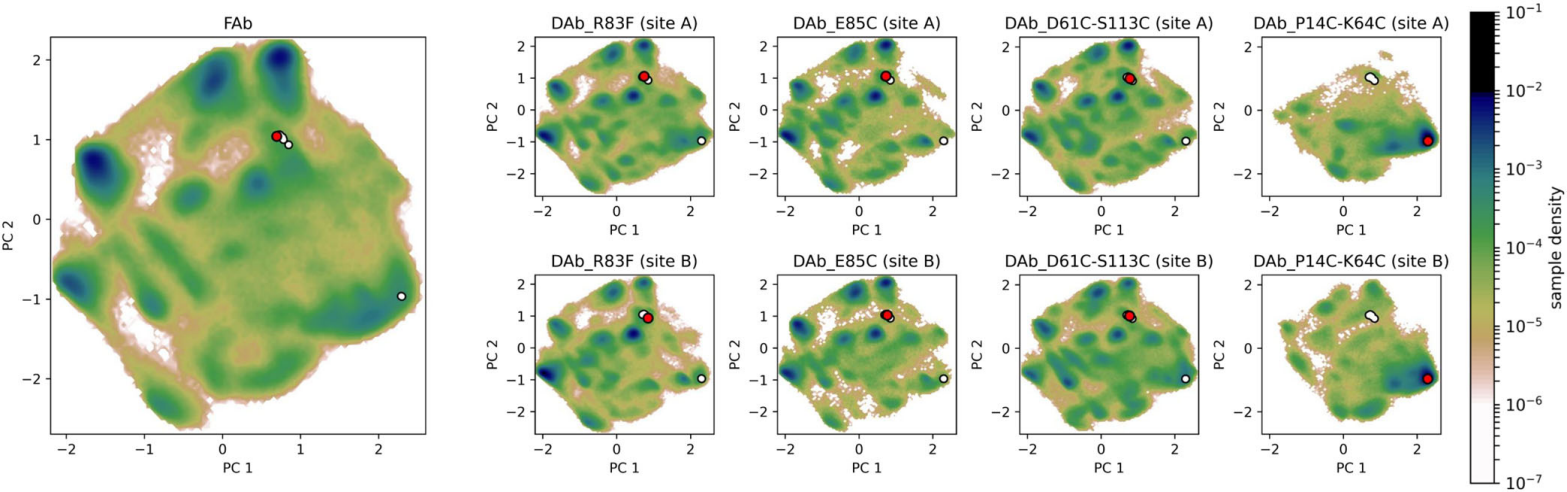
PCA plot on the CDR–H3 dihedrals: The simulation of the Fab fragment on the left (Fab) is compared to the diabodies. For each diabody, the conformational space is shown for both *F*_v_ fragments (one site per row). In each subplot the red point shows the crystal structure the corresponding simulations was started from, while the white ones represent the other crystal structures.

In contrast to the CDR‐H2 PCA plots, the conformational landscape of the CDR‐H3 loop (Figure 9) is very similar to the simulation of the Fab fragment (Fab) for most of the diabody simulations (DAb_R83F, DAb_E85C, DAb_D61C‐S113C). In these simulations, the CDR‐H3 loop forms a very complex ensemble with multiple stable conformations. However, the DAb_P14C‐K64C simulation displays a different conformational space with a single deep energetical minimum, close to its starting geometry. This conformation is also covered by the other simulations, but energetically less favored.

In most diabodies, both *F*_v_ domains (A and B) show very similar landscapes, indicating convergence of the H3 loops.

## 6 DISCUSSION

In this study, we provide an extensive in‐silico description of the structure and dynamics of diabodies.

Previously, the structure of diabodies was described in an electron micrographic^56^ and a crystallographic study.^38^ While the crystallographic approach^38^ lacks in the description of dynamics and might be distorted by crystal packing effects,^102^, ^103^, ^104^ the electron micrographic^56^ study provides the possibility to describe an ensemble in solution. However, since the latter method is based on two‐dimensional photographs of diabody‐Fab complexes, rotations of the complex make the determination of precise measures challenging.

We applied both descriptions on diabody molecular dynamics simulations and observe notably high conformational flexibility (Figures 3 and 4). This finding is in line with the electron micrographic study: we find excellent agreement in the observed (60°–170°)^56^ and simulated (60°–160°) diabody angles. The small deviations might derive from the different diabody sequences, the aforementioned inaccuracies of the experimental method, and the differences in the definition of the diabody angle.

We observe a bimodal distribution in both measures, diabody angle and diabody dihedral, similarly as in the electron micrographic study. This finding has direct influence on the mode of action: as shown by Moraga et al.,^55^ different diabody conformations can result in opposite downstream effects. Protein engineers should carefully evaluate the preferred diabody solution structures and their impact on the geometries of the ligands and, if required, make efforts to stabilize one of those conformational ensembles.

We find significant diabody dihedral deviations from the crystal structure in all systems for which we performed simulations. This indicates that crystal‐packing effects strongly affect the interdomain motions in diabodies. We therefore believe that extensive molecular dynamics simulations are more adequate to describe those conformational changes than X‐ray crystallography.

We applied the dihedral measure defined in the crystallographic study by Kim et al.^38^ on an extended set of diabody crystal structures and find that it is prone for ambiguity: the diabody crystal structures 5FCS^97^ and 5IWL^98^ share a dihedral angle of 77°, but strongly differ in structure (Figure S4). This emphasizes the need for alternative descriptions of diabody conformations, relying on multiple measures.

To address this issue, we applied a novel description of the diabody structure based on a spherical projection. By aligning one of the *F*_v_s on a reference structure, we define an absolute coordinate system. As presented in Figure 5, this metric allows to describe the diabody conformations with four measures: each one θ and ϕ angle for the not aligned *V*_H_ and *V*_L_ domain. When applied to a set of 31 crystal structures, we observe a grouping into five clusters. According to our representation, the two biggest clusters 1 and 2 are very similar. The other three clusters differ not only in their geometry, but also in their biological context: the members of cluster 3 are designed in a *V*_L_ to *V*_H_ linkage,^96^, ^97^ the members of cluster 4 show antagonistic EPO pathway activation,^55^ the sole member of cluster 5 has been covalently bound to IL2 and might, therefore, be geometrically disturbed. Interestingly, Lu et al.^32^ observed differences in expression and antigen binding of *V*_H_–*V*_L_ and *V*_L_–*V*_H_, which might be related to our finding of significantly different domain orientations.

An explanation for the strongly differing orientations of the PDB structures 4Y5X and 4Y5Y^55^ (cluster 4) from the other *V*_H_–*V*_L_ diabodies was formulated in 2016 by Kim et al.: by comparing the *F*_v_ interfaces of multiple crystal structures, they formulated the hypothesis that the amino acid at position 83 in the heavy chain is critical for the diabody interface stability. They suggest that arginines at H83 introduce a repulsion between the two heavy chains and thus make the structure more ambiguous. To test this hypothesis, Kim et al. designed mono‐ and bispecific diabodies with substituted amino acids at position H83. Additionally, they introduced disulfide bridges in the *F*_v_ interface to further restrict the diabody conformations to a defined structure.^38^

To test the hypothesis of rigidification upon the introduction of disulfide bridges, we performed extensive molecular dynamics simulations of three disulfide‐stabilized diabodies by Kim et al. (DAb_E85C, DAb_D61C‐S113C, DAb_P14C‐K64C^38^) and compared them to a simulation of a diabody with nearly identical sequence (DAb_R83F, PDB codes and sequences compared in Table 1). Unfortunately, no crystal structure without disulfide bridges, Arg on H83 and otherwise identical sequence was available on the PDB, so we cannot discuss the impact of Arg83 with our simulations.

As expected, we find a significant rigidification through the additional disulfide bonds (Figures 3 and 4). Especially the E85C mutant is strongly rigidified, even more than the two investigated double mutants. Furthermore, the interdomain orientation is significantly altered depending on the exact choice of disulfide bonds. This knowledge can be used to guide future drug development efforts by choosing disulfide bonds to optimize the shape of diabodies toward the desired binding pose.

However, one must take care when introducing disulfide bonds to avoid changing the conformation of the binding interface. In the case of the P14C‐K64C double mutant, we find a shift both in the inter‐ and interdomain conformation (Figures 6 and 7), when comparing to the other diabody simulations and the simulation of a Fab fragment. Additionally, the CDR conformations are significantly altered in this variant especially in the H3 loop. Of all CDR loops, CDR‐H3 is known to be the most critical for antigen binding.^105^

We also observe significant differences in the CDR‐H2 loop movements (Figure 8) in all diabody systems, when comparing to a Fab fragment simulation. Interestingly, the P14C‐K64C mutant has the most similar CDR‐H2 conformational ensemble compared to the Fab fragment, while it differs most in all other measures. Of all CDR loops, H2 lies the closest to the artificial *F*_v_–*F*_v_ interface and might thus be influenced by the diabody format. This observation should be verified with future studies.

In general, the CDR loop conformational analysis results should be handled with care since they only provide insights of loop movements in the micro‐ to millisecond timescale. Although this timescale was previously shown to be most critical in antibody CDR loop movements,^10^, ^11^, ^13^, ^106^, ^107^ we find slight inconsistencies when comparing the CDR loops between both sites of the diabody. This implicates that larger‐scale movements might be involved in the dynamics of diabodies.

The most significant differences between site A and B are found in the H2 loops of DAb_R83F. This diabody variant is the only one without stabilizing disulfide bonds in our dataset and is thus expected to be the most flexible. Additionally, the two X‐ray structures differ significantly, which could indicate crystal packing effects and a bias in the MD‐derived probability of site B. The differences might also derive from insufficient sampling. Either hypothesis could be verified by extending the sampling until convergence of the two diabody sites.

In summary, our findings are relevant for future drug development efforts, by highlighting that the CDR‐H2 loop movements might be altered by the diabody format, when comparing to an antibody. Moreover, it shows that the P14C_K64C mutation should be avoided when it is expected that the H3 loop affects the binding properties.

## 7 CONCLUSION

In this study, we analyze the structure of diabodies both in crystal structures and in solution. We introduce a new measure, based on a spherical projection, and observe a clustering of 31 crystal structures into 5 major clusters, in line with their biological context.

We find high conformational flexibility in the relative orientation of the two *F*_v_ domains in molecular dynamics simulations and observe rigidifying effects when comparing diabodies with and without interdomain disulfide bonds. Furthermore, we compare the *V*_H_–*V*_L_ orientations and CDR conformations to the simulation of a Fab fragment, and find mostly consistent dynamics, indicating similar antigen binding properties with the highest deviations in the CDR‐H2 loop movements. However, the diabody variant with two disulfide bonds at P14C and K64C shows a shift in most of our measures compared to the Fab fragment, including the CDR‐H3 loop movements. This finding underlines the need for careful validation when designing stabilizing disulfide bridges, as their location might influence the antigen binding properties.

## AUTHOR CONTRIBUTIONS

**Barbara A. Math:** Investigation; writing – original draft; visualization; methodology; formal analysis. **Franz Waibl:** Writing – review and editing; software. **Leonida M. Lamp:** Conceptualization; writing – review and editing. **Monica L. Fernández‐Quintero:** Writing – review and editing; conceptualization; supervision; project administration; funding acquisition. **Klaus R. Liedl:** Supervision; resources; project administration; writing – review and editing; conceptualization; funding acquisition.

## CONFLICT OF INTEREST STATEMENT

The authors declare no conflict of interest.

## Supporting information


**FIGURE S1.** Representation of the dataset, consisting of four diabodies (5GRW, 5GRX, 5GRY, 5GRZ^1^) and a Fab fragment (2V7N^2^). For the diabody variants, an insert is shown, displaying the location of the stabilizing disulfide bonds in yellow and the residue at H83 in pink.
**FIGURE S2.** Overview on the structural effect of the dihedral angle: Random simulation frames of the seeded simulations of DAb_R83F with dihedral angles between 0° and 105° are shown.
**FIGURE S3.** Overview on the structural effect of the diabody angle: Random simulation frames of the seeded simulations of DAb_R83F with diabody angles between 60° and 155° are shown.
**FIGURE S4.** Cartoon representation of two crystal structures (PDB codes 5FCS^3^ and 5IWL^4^), sharing the same dihedral angle of 77°. The two structures are aligned on one of the *F*_v_s.

Supporting Information.

## Data Availability

The authors confirm that the data supporting the findings of this study are available within the article and its supplementary materials.
