# Supplementary material for: Cross‐linking disulfide bonds govern solution structures of diabodies
Source: Proteins. 2023 Jun 28;91(9):1316–28. doi: 10.1002/prot.26509 (PMC10952579; doi:10.1002/prot.26509)
Supplement: Supplementary file 1 — FIGURE S1. Representation of the dataset, consisting of four diabodies (5GRW, 5GRX, 5GRY, 5GRZ 1 ) and a Fab fragment (2V7N 2 ). For the diabody variants, an insert is shown, displaying the location of the stabilizing disulfide bonds in yellow and the residue at H83 in pink. FIGURE S2. Overview on the structural effect of the dihedral angle: Random simulation frames of the seeded simulations of DAb_R83F with dihedral angles between 0° and 105° are shown. FIGURE S3. Overview on the structural effect of the diabody angle: Random simulation frames of the seeded simulations of DAb_R83F with diabody angles between 60° and 155° are shown. FIGURE S4. Cartoon representation of two crystal structures (PDB codes 5FCS 3 and 5IWL 4 ), sharing the same dihedral angle of 77°. The two structures are aligned on one of the F vs. [file PROT-91-1316-s002.pdf]

Supporting Information

Cross-linking disulfide bonds govern solution structures of diabodies

Barbara A. Math, Franz Waibl, Leonida M. Lamp, Monica L. Fernández-Quintero, Klaus R. Liedl

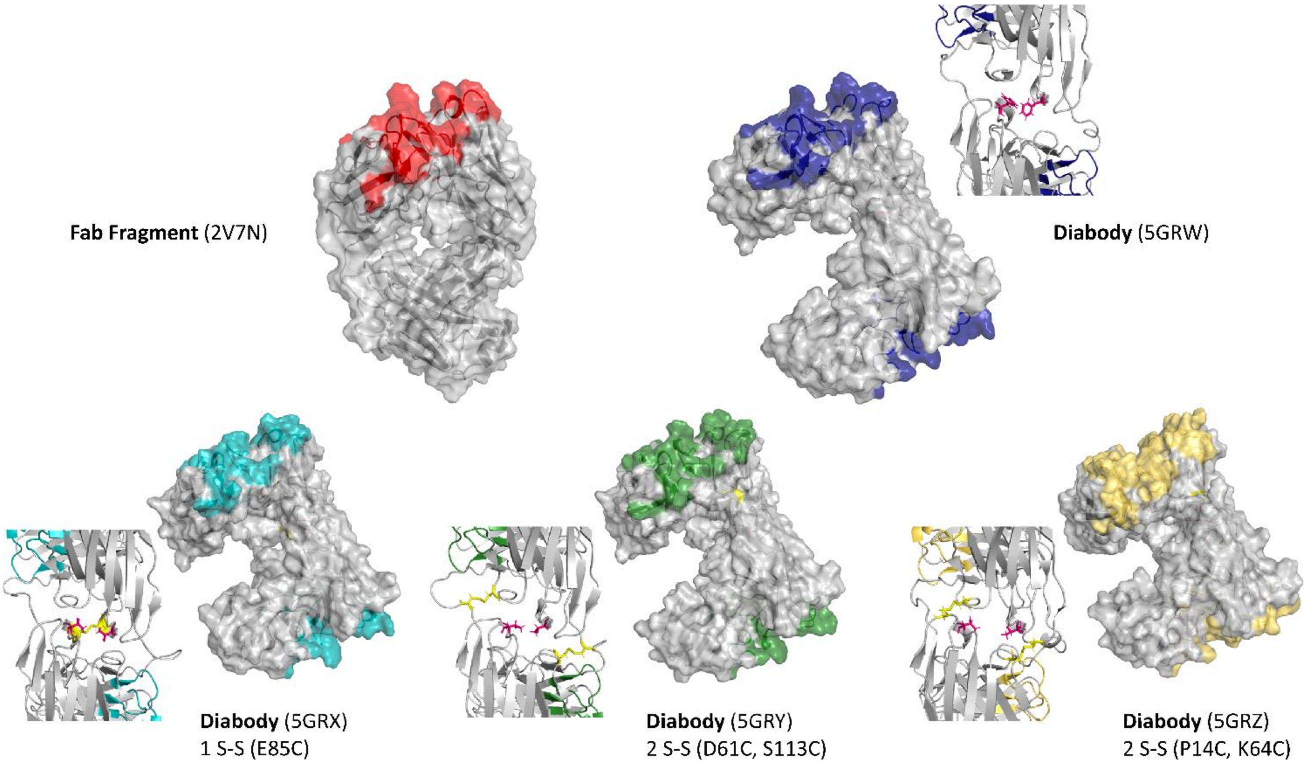

SI Figure 1: Representation of the dataset, consisting of four diabodies (5GRW, 5GRX, 5GRY, 5GRZ [1]) and a Fab fragment (2V7N [2]). For the diabody variants an insert is shown, displaying the location of the stabilizing disulfide bonds in yellow and the residue at H83 in pink.

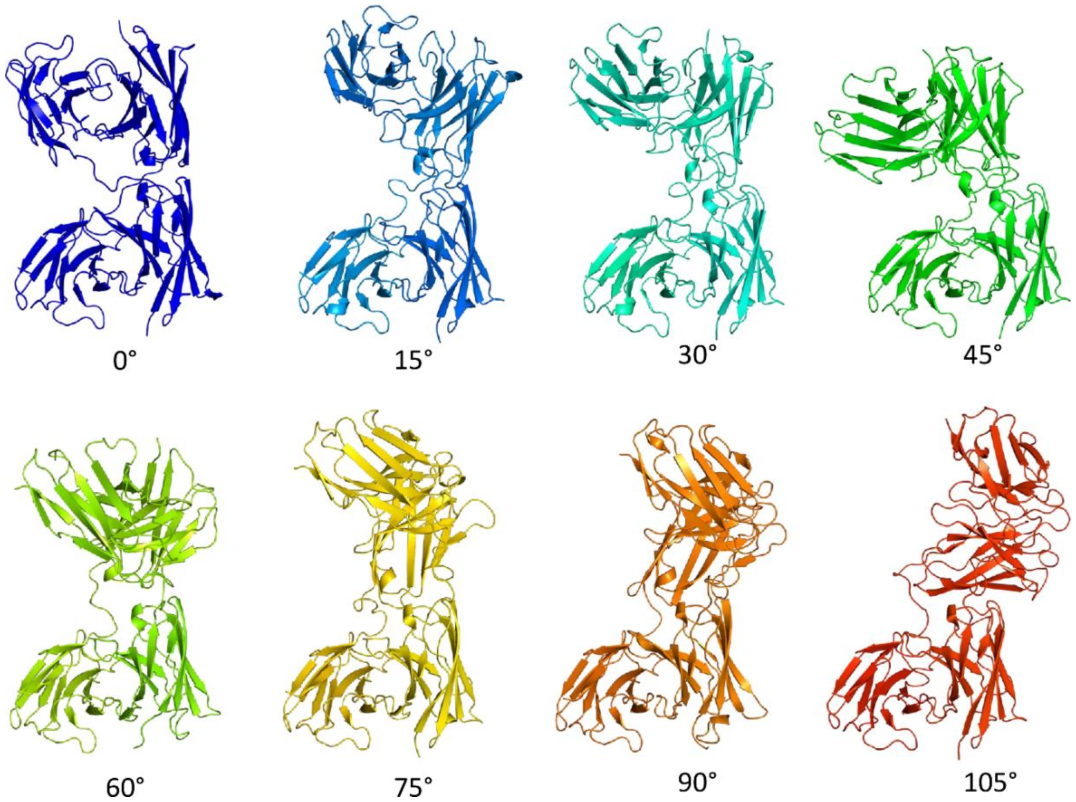

SI Figure 2: Overview on the structural effect of the dihedral angle: Random simulation frames of the seeded simulations of DAb_R83F with dihedral angles between 0 and 105° are shown.

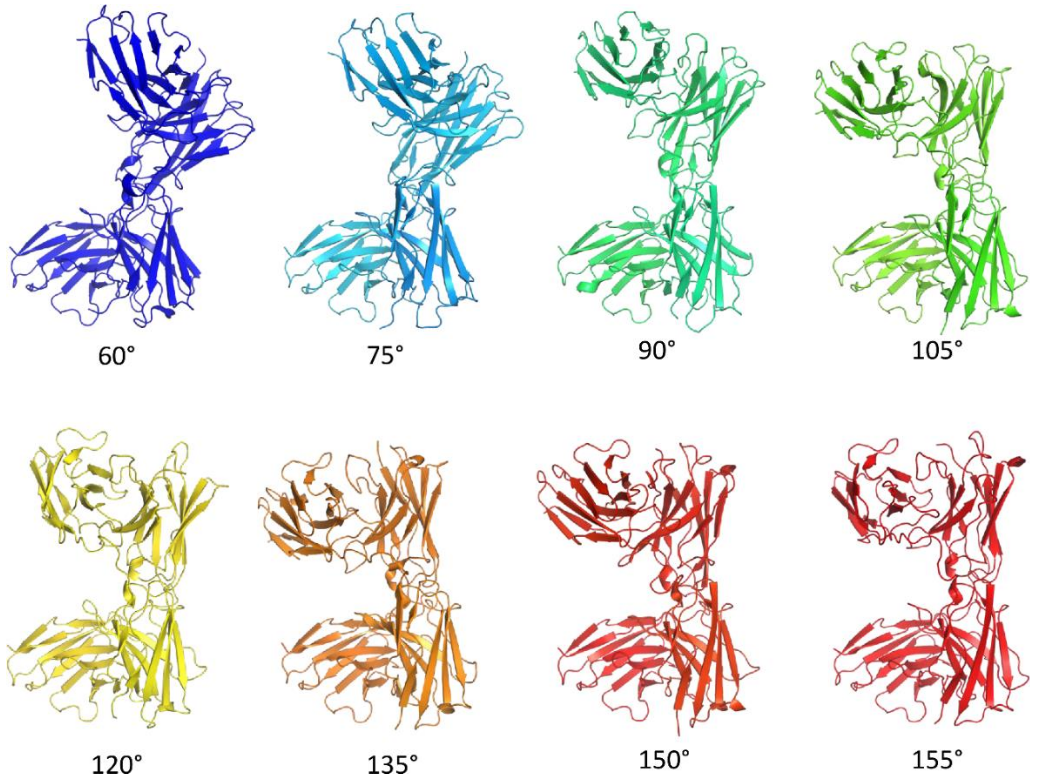

SI Figure 3: Overview on the structural effect of the diabody angle: Random simulation frames of the seeded simulations of DAb_R83F with diabody angles between 60 and 155° are shown.

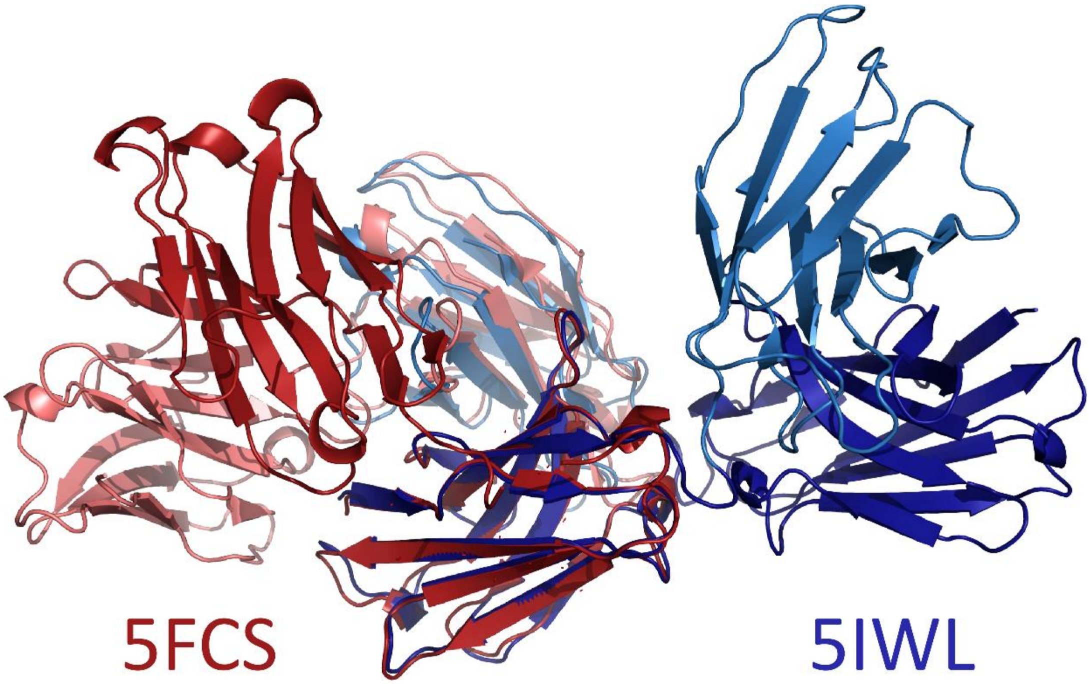

SI Figure 4: Cartoon representation of two crystal structures (pdb codes 5FCS[3] and 5IWL[4]), sharing the same dihedral angle of 77°. The two structures are aligned on one of the F_v*s.*

References

1. DeKosky, B.J., et al., *Large-scale sequence and structural comparisons of human naive and antigen-experienced antibody repertoires.* Proceedings of the National Academy of Sciences, 2016. **113**(19): p. E2636-E2645.
2. Frey, D., et al., *Structure of the recombinant antibody Fab fragment f3p4.* Acta Crystallographica Section D: Biological Crystallography, 2008. **64**(6): p. 636-643.
3. Root, A.R., et al., *Development of PF-06671008, a highly potent anti-P-cadherin/anti-CD3 bispecific DART molecule with extended half-life for the treatment of cancer.* Antibodies, 2016. **5**(1): p. 6.
4. Weiskopf, K., et al., *CD47-blocking immunotherapies stimulate macrophage-mediated destruction of small-cell lung cancer.* The Journal of clinical investigation, 2016. **126**(7): p. 2610-2620.
